# Impact of public hospital restructuring on the admission of elderly residents in Japan: a regional population-based study

**DOI:** 10.1186/s12913-026-14388-3

**Published:** 2026-04-02

**Authors:** Kenji Kishimoto, Susumu Kunisawa, Yuichi Imanaka

**Affiliations:** 1 Department of Healthcare Economics and Quality Management, Graduate School of Medicine, Kyoto University, Kyoto, Japan; 2 Department of Health Security System, Center for Health Security, Graduate School of Medicine, Kyoto University, Yoshida Konoe-cho, Sakyo-ku, Kyoto, 606-8501 Japan

**Keywords:** Hospital restructuring, Regional healthcare, Elderly, Hospitalization

## Abstract

**Background:**

Although many countries have reformed public hospitals to reduce the imbalance of resources, there is limited information on the impact of hospital restructuring on communities and the regional healthcare system. The present study aimed to examine the effects of public hospital restructuring on the admission of elderly residents.

**Methods:**

We analyzed a public hospital restructuring case in a secondary medical service area (SMSA) in Japan using administrative claims data. All consecutive patients aged 65 years or older who resided in the SMSA and were admitted to a hospital or visited a hospital between 36 months prior and 48 months after the restructuring were included. Effects of the restructuring on monthly admissions within the SMSA were evaluated using a segmented Poisson regression model and interrupted time-series analysis.

**Results:**

A total of 58,929 admissions were analyzed. After the restructuring, admissions within the SMSA increased and reached the same level of admissions outside the SMSA. Interrupted time-series analyses revealed level (incidence rate ratio (IRR) 1.097, 95% confidence intervals (CI) 1.039–1.159, + 10.3% in two months) and slope (IRR 1.005, 95% CI 1.003–1.007, + 7 per month) changes in monthly admissions within the SMSA after the restructuring.

**Conclusions:**

Monthly admissions of elderly residents within the SMSA increased after hospital restructuring, underscoring the importance of examining admissions within the neighborhood when assessing the impact of public hospital restructuring.

**Supplementary Information:**

The online version contains supplementary material available at 10.1186/s12913-026-14388-3.

## Background

Hospitals are expected to provide safe and effective healthcare services as a major component of the healthcare system [1]. Hospital characteristics include institutional size, number of beds, technology status, teaching status, geographic location, and ownership. Among these, ownership structure is a key determinant of hospital performance [2, 3]. Public hospitals often face difficulties such as limited resources and financial distress [4–6], and reforming them is known to be inherently difficult [7]. Many countries have reformed public hospitals with the intent of reducing resource imbalances. Previous reports suggest that public hospital restructuring may alleviate the mismatch of resources in the healthcare system [8–11]. These studies often adopt qualitative approaches and lack objective evidence to quantify the effects of restructuring [10, 11].

Population aging and regional disparities can contribute to imbalances in the medical resource distribution [12–14]. Aging of the population increases the need for healthcare services by community-dwelling elderly people [15, 16]. Underpopulated areas which are at risk of lacking human resources generally have a large elderly population. While Japan has the highest percentage of the population aged ≥ 65 years in the world (29.1% in October 2023), there are regional disparities in the proportion of this population [17]. Marked regional disparities in healthcare utilization for elderly patients have also been reported in a Japanese nationwide study [18]. The increasing demand for healthcare is accompanied by the pressing need to optimize the healthcare system in line with regional characteristics.

Only a few studies have focused on the effects of hospital restructuring on communities and the regional healthcare system. Previous studies examining the impact of service reconfigurations in hospitals revealed changes in admissions, length of stay, and mortality in regional settings [19, 20]. Given that population aging and regional disparities are common and significant problems, examining the impact of public hospital restructuring on hospital use by elderly residents is meaningful and informative. We propose a hypothesis that public hospital restructuring can affect the local capacity for hospital admissions, resulting in a change in admissions within the region after the restructuring. We focused on the restructuring of public hospitals rather than that of private hospitals from the perspective that the former would be more feasible to conduct in line with regional needs. To this end, the present study aimed to examine the impact of public hospital restructuring on elderly hospital admissions in Japan.

## Methods

### Patient and public involvement

Patients and/or the public were not involved in the design, conduct, reporting or dissemination plans of this study.

### Data source

We analyzed hospital admissions and outpatient visits over time using administrative claims data obtained from the National Health Insurance and Late-Stage Medical Care Scheme for the Elderly of a prefecture in Japan. National Health Insurance is one of the two major health insurance programs in Japan [21], covering 70.8% of people aged 65–74 years in the prefecture in 2019. The Late-Stage Medical Care Scheme for the Elderly is the only healthcare insurance program available to those aged ≥ 75 years in Japan [21]. Claims data include patient-level information on hospital codes, patient demographics, residence area, admission and discharge dates, diagnoses based on International Classification of Diseases 10th revision codes, and claims for medical services. The number of people insured through National Health Insurance and the Late-Stage Medical Care Scheme for the Elderly in the prefecture was obtained from the Portal Site of the Official Statistics of Japan (e-Stat) [22].

### Hospital restructuring

There are five secondary medical service areas (SMSAs) in the prefecture. An SMSA is a sub-prefectural region defined as a medical unit that evaluates demand and supply of health resources [23, 24]. Hospital restructuring was conducted in the largest SMSA of the prefecture covering more than 60% of the prefectural area, where 5% of the prefectural population lives. The background information on the SMSA and population composition during the study period is shown in Supplemental Tables 1 and 2. This SMSA has been facing the problems of aging and a declining population. Although there were three public acute care hospitals with a total 572 beds (572 acute care beds) and one private hospital with 138 beds (overall 710 beds) in the SMSA before the restructuring, the low capacity to provide acute care led to a situation in which more than half of the residents in the SMSA who required hospitalization were admitted to other SMSAs. In addition, half of the residents in the SMSA who needed urgent care were transferred to other SMSAs because the emergency department lacked sufficient human and medical resources to implement urgent care. Thus, hospital restructuring was performed in April 2016, to focus human and medical resources on providing acute care for residents in the SMSA. Through this restructuring, a new public general medical center and two public chronic care hospitals were formed, reducing the number of public hospital beds from 572 to 399 (327 acute care beds and 72 chronic care beds) (overall 537 beds in the SMSA) (Supplementary Fig. 1) and increasing the absolute number of doctors by 1.37-fold. The new chronic care hospitals are located in the same place as the former acute care hospitals before the restructuring, and the new medical center was moved nearly 5 km from the location of the former acute care hospital. Ownership of hospitals was transferred from the prefecture and municipalities to a public medical organization, which consisted of the municipalities in the SMSA and the prefecture. There was no structural change for the private hospital and there were no inpatient clinics during the study period.

### Study population

All consecutive patients aged 65 years or older who resided in the SMSA and were admitted to a hospital or visited a hospital between 36 months before and 48 months after the hospital restructuring were included in the study. Age groups used in the present study were 65–74 years, 75–84 years, and ≥ 85 years, and the two time periods assessed were the pre-restructuring period (36 months) and the post-restructuring period (48 months).

### Statistical analysis

This study was planned and performed after the post-restructuring period. The primary outcome measure was the monthly incidence of admissions within the SMSA. Secondary outcome measures were the monthly incidence of outpatient visits, including both routine care and urgent care and the monthly incidence of inpatient surgeries within the SMSA. Data from repeated admissions of or visits by the same patient were included in the analysis. The incidence is presented as monthly number per million insured population in the SMSA. Monthly age-standardized incidence was calculated using the insured SMSA population in the first year of the pre-restructuring period. Time-series results of the primary outcome measure were presented in total and by age group. We evaluated whether the hospital restructuring had an impact on the primary outcome measure using interrupted time-series analysis [25] to compare level and slope changes in monthly admissions between the pre-restructuring and post-restructuring periods. Hospital restructuring was treated as the intervention and coded as a binary variable. The monthly number of admissions within the SMSA and the intervention were considered dependent and independent variables, respectively. As the dependent variable was a count, a segmented Poisson regression model was used [25]. The time (in month units) since the start of the study period, interaction term of the intervention and post-intervention time (in month units), log number of insured as the offset term, and Fourier terms for seasonality were also included in the model. Incidence rate ratios (IRRs) and 95% confidence intervals (CIs) were calculated from the model. Model adequacy was assessed using standardized residual plots. Autocorrelation was evaluated using an autocorrelation function and the Cumby–Huizinga test. To avoid the effect of the regulation of admissions before and after the hospital restructuring, the transition period two months before and after the intervention was excluded from the primary analysis [26, 27]. A sensitivity analysis was subsequently performed using all time points. The validity of the transition period was assessed by visual inspection and sensitivity analysis.

*P* < 0.05 was considered statistically significant, and all tests were two-tailed. Statistical analyses were conducted with Stata/SE version 16.1 (StataCorp. College Station, TX, USA).

### Ethical considerations

The present study was approved by the Ethics Committee of the Graduate School of Medicine, Kyoto University (approval number: R0438), and conducted in accordance with the Ethical Guidelines for Medical and Health Research Involving Human Subjects of the Ministry of Health, Labour and Welfare, Japan.

## Results

During the study period, 58,929 admissions and 2,364,096 outpatient visits of patients who resided in the SMSA were identified. Patient demographics are summarized in Table 1. The median age of inpatients and outpatients was 80 (interquartile range 74–86) years and 78 (72–84) years, respectively. The 75–84 years age group had the most number of inpatients and outpatients. Proportions of patients admitted within the SMSA during the pre-restructuring and post-restructuring periods were 43.1% and 51.8%, respectively. The distributions of length of stay were similar between the two periods.

**Table 1:** Patient characteristics

	Admissions			Outpatient visits		
	Overall period (*N* = 58,929)	Pre-restructuring period (*n* = 24,190)	Post-restructuring period (*n* = 34,739)	Overall period (*N* = 2,364,096)	Pre-restructuring period (*n* = 1,008,981)	Post-restructuring period (*n* = 1,355,115)
Median age, years (IQR)	80 (74–86)	80 (74–86)	81 (75–87)	78 (72–84)	78 (72–83)	78 (72–84)
Age group, n (%)						
65–74 years	14,926 (25.3)	6,409 (26.5)	8,517 (24.5)	777,934 (32.9)	334,629 (33.2)	443,305 (32.7)
75–84 years	25,255 (42.9)	10,706 (44.3)	14,549 (41.9)	1,057,642 (44.7)	463,128 (45.9)	594,514 (43.9)
≥ 85 years	18,748 (31.8)	7,075 (29.2)	11,673 (33.6)	528,520 (22.4)	211,224 (20.9)	317,296 (23.4)
Female, n (%)	29,975 (50.9)	12,065 (49.9)	17,910 (51.6)	1,388,874 (58.7)	591,979 (58.7)	796,895 (58.8)
Location of medical institution, n (%)						
Within SMSA	28,435 (48.3)	10,432 (43.1)	18,003 (51.8)	1,682,030 (71.1)	722,783 (71.6)	959,247 (70.8)
Outside SMSA	30,494 (51.7)	13,758 (56.9)	16,736 (48.2)	682,066 (28.9)	286,198 (28.4)	395,868 (29.2)
Median length of stay, days (IQR)	12 (4–28)	12 (4–30)	12 (4–27)	-	-	-

Abbreviations: IQR, interquartile range; SMSA, secondary medical service area

### Time-series of outcomes

**Fig. 1:**
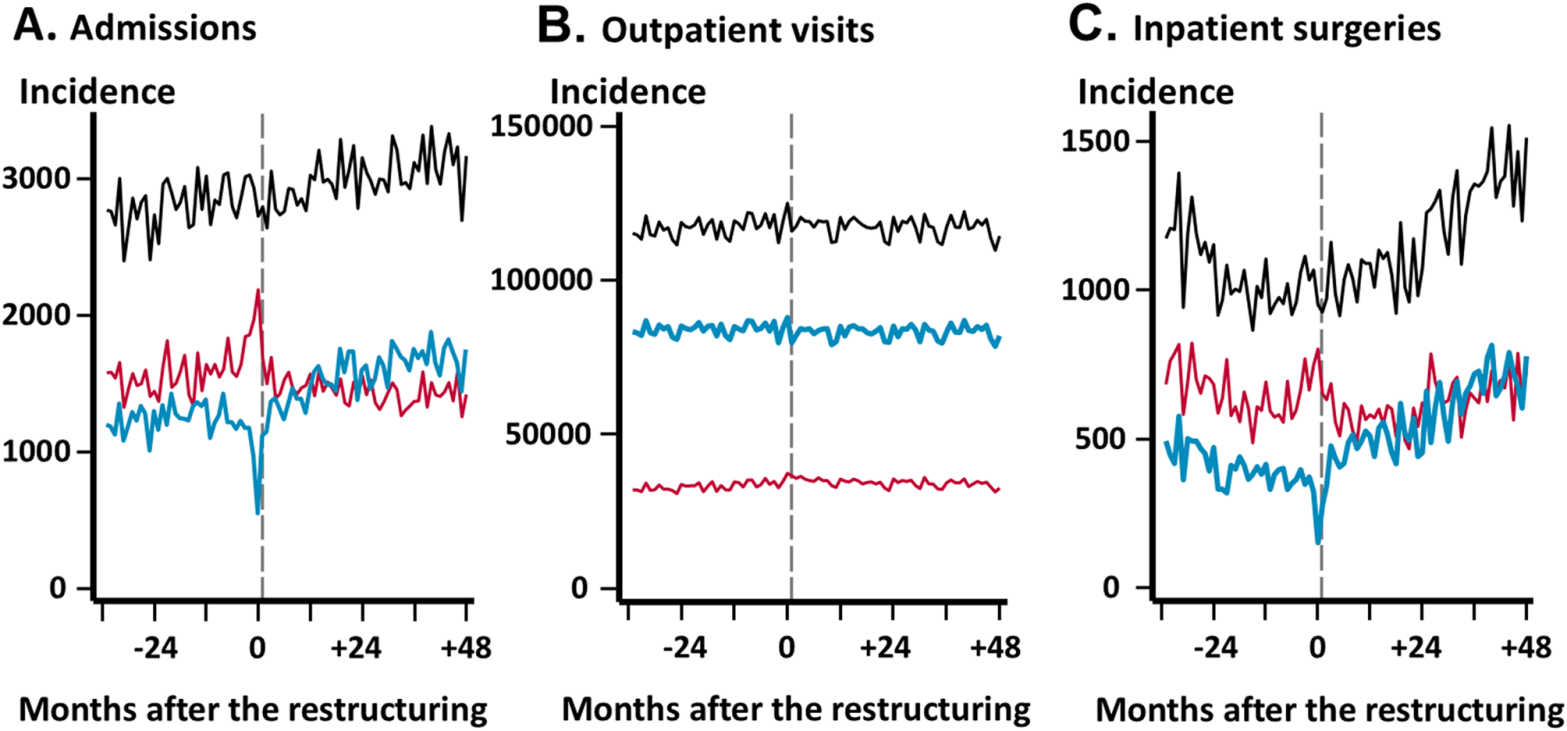
Time trends in the monthly incidence per million insured population before and after the hospital restructuring. Gray vertical dashed lines indicate the time point at which the hospital restructuring occurred. Blue lines represent outcomes within the secondary medical service area (SMSA). Red lines represent outcomes outside the SMSA. Black lines represent overall outcomes. (**A**) Admissions; (**B**) Outpatient visits; and (**C**) Inpatient surgeries

Figure 1 shows time trends in the monthly incidence per million insured population before and after the hospital restructuring. Overall, a secular increasing trend in admissions was observed throughout the study period (Fig. 1A, bold dashed line). Before the hospital restructuring, more patients were admitted outside the SMSA than within the SMSA (Fig. 1A, thin dashed line). Admissions within the SMSA were markedly reduced at two months before the hospital restructuring, whereas admissions outside the SMSA increased transiently. After the hospital restructuring, admissions within the SMSA increased gradually to reach the same level of admissions outside the SMSA (Fig. 1A, bold black line). A consistent increase in age-standardized incidence of admissions within the SMSA was also observed in the post-restructuring period (Table 2). There were no obvious changes in trends of outpatient visits (Fig. 1B). The number of inpatient surgeries decreased in the pre-restructuring period and then increased in the post-restructuring period, reflecting the change in surgical capacity through hospital restructuring (Fig. 1C).

**Table 2:** Average monthly age-standardized incidence of admissions

	Pre-restructuring period (3 years)			Post-restructuring period (4 years)				
	First year	Second year	Last year	First year	Second year	Third year	Last year	
Overall admission (per million)	2,729	2,844	2,869	2,830	3,035	3,005	3,083	
Within SMSA (per million)	1,214	1,293	1,139	1,319	1,576	1,621	1,659	
Outside SMSA (per million)	1,515	1,551	1,730	1,511	1,459	1,384	1,424	

Abbreviations: SMSA, secondary medical service area

**Fig. 2:**
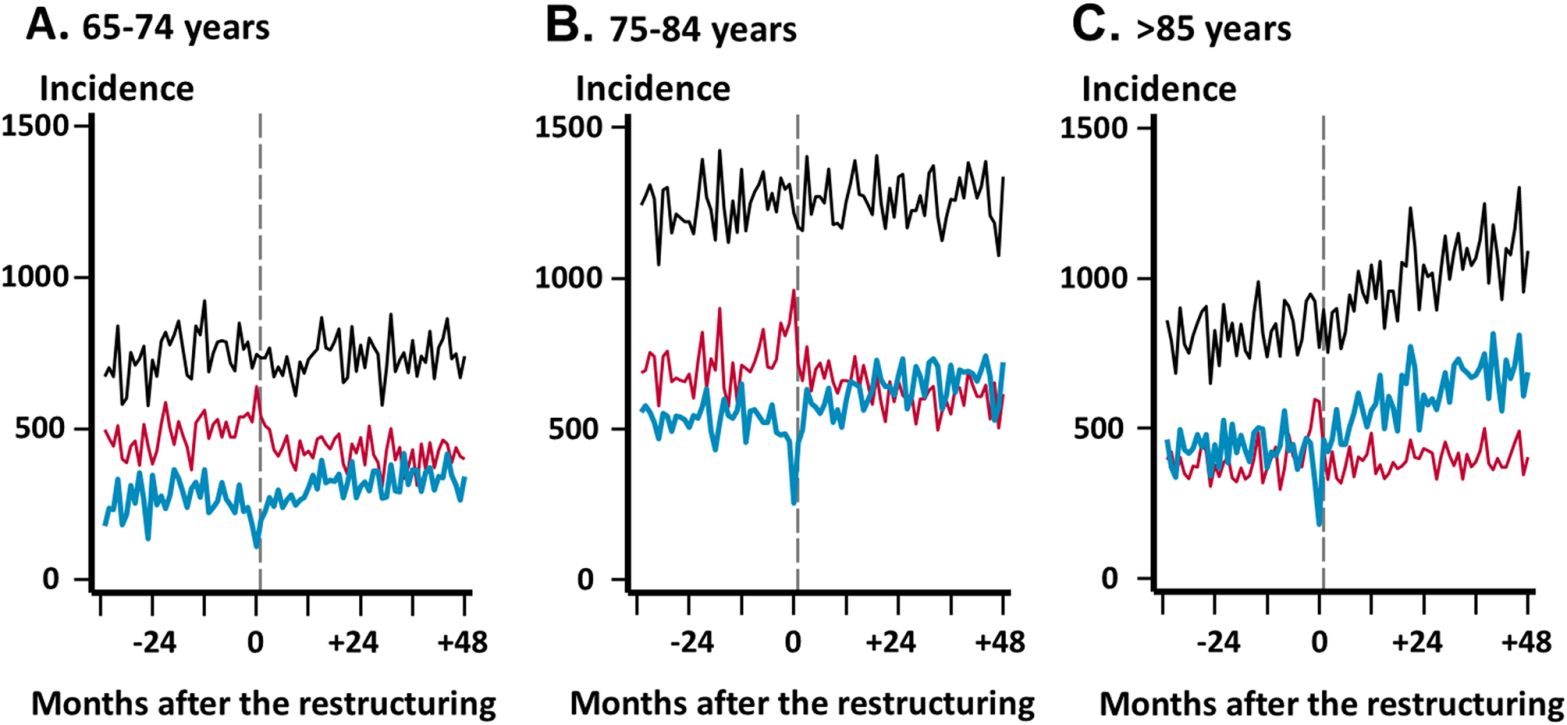
Time trends in the monthly admissions per million insured population by age group. Gray vertical dashed lines indicate the time point at which hospital restructuring occurred. Blue lines represent admissions within the secondary medical service area (SMSA). Red lines represent admissions outside the SMSA. Black lines represent overall admissions. (**A**) Aged 65–74 years; (**B**) Aged 75–84 years; and (**C**) Aged ≥ 85 years

Monthly time-series of admissions by age group are shown in Fig. 2. In the pre-restructuring period, admissions within the SMSA were lower than admissions outside the SMSA among the 65–74 years age group and 75–84 years age group (Fig. 2A and B), while admissions within and outside the SMSA were similar in the ≥ 85 years age group (Fig. 2C). The increasing trend in admissions within the SMSA during the post-restructuring period was most pronounced in the ≥ 85 years age group (Fig. 2C).

### Impact of hospital restructuring on admissions within the SMSA

**Fig. 3:**
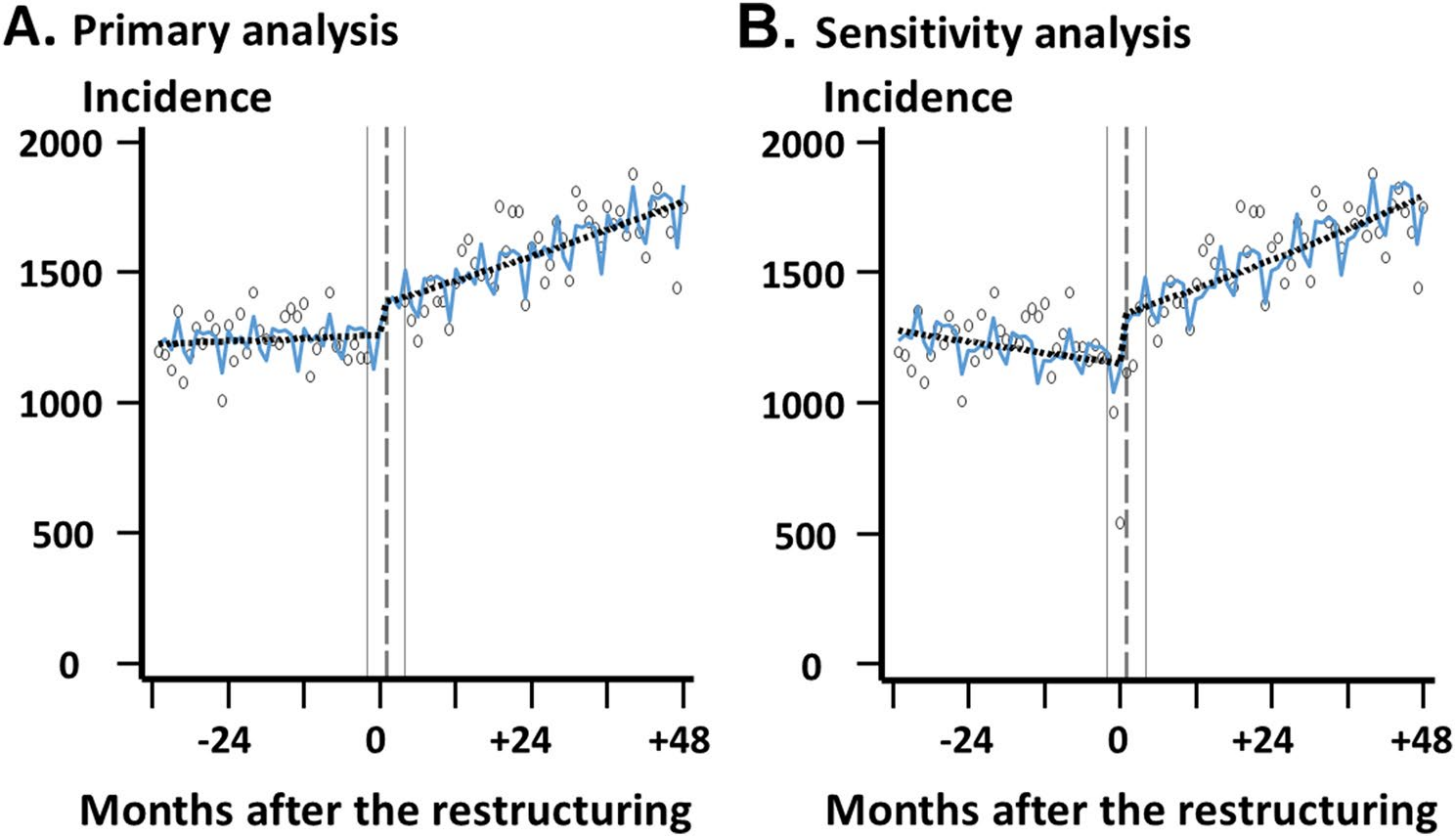
Results of interrupted time-series analyses. Gray vertical dashed lines indicate the time point at which the hospital restructuring occurred. Gray vertical thin lines indicate the period between two months before and two months after the restructuring. Scattered points represent actual monthly admissions within the secondary medical service area per million insured population at each time point. Black dot lines represent estimated incidence trends without seasonality. Blue curves represent estimated incidence trends with seasonality. (**A**) Primary analysis excluding the period two months before and after the restructuring; (**B**) Sensitivity analysis including all time points

Table 3; Fig. 3 depict the results of interrupted time-series analyses assessing the level and slope changes in monthly admissions within the SMSA per million insured population between the two periods. Compared with two months before hospital restructuring, the estimated incidence of admissions within the SMSA markedly increased from 1,257 to 1,386 (difference, + 129 (+ 10.3%)) at two months after the restructuring, indicating a significant level change (IRR 1.097, 95% CI 1.039–1.159) (Fig. 3A). No obvious secular trend was found in the pre-restructuring period. After the hospital restructuring, a positive slope change (IRR 1.005, 95% CI 1.003–1.007) was observed, resulting in an increasing trend (+ 7 admissions per month (+ 98 admissions annually) within the SMSA).

**Table 3:** Interrupted time-series analyses on the impact of hospital restructuring on monthly incidence of admissions within the SMSA

	Primary analysis			Sensitivity analysis		
	IRR	95% CI	*P* value	IRR	95% CI	*P* value
Pre-restructuring slope	1.001	0.999–1.003	0.602	0.997	0.995–0.999	0.005
Level change	1.097	1.039–1.159	0.001	1.151	1.094–1.210	< 0.001
Slope change	1.005	1.003–1.007	< 0.001	1.009	1.007–1.011	< 0.001
Intercept	1225	1176–1276	< 0.001	1274	1224–1325	< 0.001

Abbreviations: CI, confidence interval; IRR, incidence rate ratio; SMSA, secondary medical service area

Sensitivity analysis with all time points showed a decreasing secular trend in the pre-restructuring period (Fig. 3B), leading to a larger difference in estimated admissions (difference, + 191 (+ 16.6%)) between one month before and one month after the restructuring. Level and slope changes were also pronounced in the sensitivity analysis.

## Discussion

The present study demonstrated the impact that public hospital restructuring had on the admission of elderly residents in a prefecture of Japan. This public hospital restructuring had three aspects: centralization of acute care, increase in chronic care beds with a reduction of total hospital beds, and increase in the number of doctors. After the hospital restructuring, monthly admissions markedly increased within the SMSA. Moreover, interrupted time-series analyses revealed remarkable level and slope changes in monthly admissions within the SMSA after the restructuring. Notably, the effect size in monthly admissions was clinically significant. These changes increased the proportion of patients who could receive in-hospital care within the SMSA, in excess of the proportion of patients outside the SMSA. These results suggest that the restructuring may have increased the local capacity of hospital admissions for elderly residents by focusing resources. The increase in admissions might be explained by more efficient bed utilization and management since the total number of beds was lower after the restructuring. The secular increase in overall admissions of elderly residents was consistent with previous reports [28–30]. In addition, the magnitude of the impact after the restructuring was most pronounced in the highest age group, suggesting that the need for hospitalizations in the community may be particularly relevant in the advanced elderly population. Considering that hospitalization of very elderly patients has been reported to have distinctive features, including diagnoses and comorbidities [29, 30], our results may further imply the existence of a previously unmet need for hospitalization in this population and the need to shift regional healthcare services to fit the specificities of regional population aging.

Previous studies have investigated the effect of hospital reorganization, mergers, and restructuring from several aspects such as clinical outcomes [19], institutional activity [31], medical costs [32], accessibility [33], and staff outcomes [34]. Recent studies have investigated changes in quality of care after hospital reforms [35–37], with inconclusive results on quality improvement. However, there is considerable heterogeneity across these studies in terms of how the indicators were defined, methods of measuring effects, and follow-up periods, underscoring the need for clear indicators and evaluation framework to assess the impact of hospital reforms [38]. Furthermore, although hospital reforms can influence patients and employees, little is known about their impact on communities. Since hospitals are important repositories for specialized clinical knowledge and advanced technology [34], changes in hospital service use are relevant to communities and regional healthcare systems.

Population-based admission rates have been used as indicators of regional healthcare services [39–41]. Admission rates vary widely for many reasons, including differences in hospital factors as well as demographic, socioeconomic, and geographic factors [42, 43]. A number of recent studies used population-based admission rate as a major outcome measure to evaluate the effect of regional healthcare system reforms [20, 44]. For instance, a population-based study from Scotland described the heterogeneous changes in emergency admission rate after episodes of inpatient facility reconfiguration, finding that several factors drove admissions after reconfiguration [20]. Our results also imply that the trend in hospital admissions within the community may be useful as a potential indicator for assessing hospital restructuring.

The strength of the present study lies in the population-based approach and relatively long study period. Our data included more than 70% of those aged 65 years and older and all residents aged 75 years and older. Although we could not assess the impact of hospital restructuring among those aged 65–74 years who were not covered by National Health Insurance, the influence of health insurance type on hospital admissions appears to be minimal given that Japan has a universal health coverage system and the fees paid to hospitals and physicians for health insurance coverage are uniform throughout the country [45]. Another strength is that we assessed post-restructuring changes both within and outside the SMSA, enabling us to understand the trends more comprehensively.

The present study also has several limitations. First, due to the nature of the database used, we lacked information regarding hospital accessibility. Since accessibility is associated with healthcare use [46, 47], this limitation makes it difficult to distinguish the effect of hospital restructuring itself from the effect of changes to hospital accessibility in the SMSA. Second, we did not assess socioeconomic variables. Previous studies found that socioeconomic factors are associated with the risk of physician visits and hospitalizations [48, 49]. During the study period, however, we did not identify any social events which may have significantly changed the socioeconomic distribution of the SMSA. Third, some changes in healthcare services outside the SMSA during the study period could have introduced potential unmeasured confounding. For instance, an intervention other than restructuring during the same period could have violated the assumption behind the interrupted time series analysis. Nevertheless, the impact of this limitation appears to be mitigated, given the relatively consistent trends in overall admissions and outpatient visits throughout the study period. Fourth, our results may not be generalizable to other settings which have differing geographic, socioeconomic, and healthcare characteristics. Fifth, there was a lack of information on the quality of care. Future studies are warranted to evaluate changes in patient-oriented outcomes following hospital restructuring. Despite these limitations, our findings provide useful insight for the evaluation of hospital restructuring in regional healthcare systems.

## Conclusions

In conclusion, we assessed the effects of restructuring public hospitals on elderly hospital admissions in a prefecture of Japan and found a marked increase in admissions within the SMSA after the restructuring. Marked level and slope changes in monthly admissions within the SMSA after the restructuring were identified using interrupted time-series analyses. These findings suggest that the restructuring led to improvements in the local capacity for elderly hospital admissions and imply that the trend in hospital admissions within the community may be a potential indicator for evaluating public hospital restructuring. Further studies to investigate optimal strategies for evaluating the impact of hospital reforms on communities and regional healthcare systems are warranted.

## Supplementary Information

Below is the link to the electronic supplementary material.

Supplementary Material 1: Supplementary Fig. 1. Hospital restructuring in the secondary medical service area.

Supplementary Material 2

## Data Availability

The datasets analyzed during the current study are available from the corresponding author on reasonable request.

## Abbreviations

CI: Confidence interval
IRR: Incidence rate ratios
SMSA: Secondary medical service areas
