# Supplementary figures and images for: Impact of public hospital restructuring on the admission of elderly residents in Japan: a regional population-based study
Source: BMC Health Serv Res. 2026 Apr 2;26:686. doi: 10.1186/s12913-026-14388-3 (PMC13169532; doi:10.1186/s12913-026-14388-3)

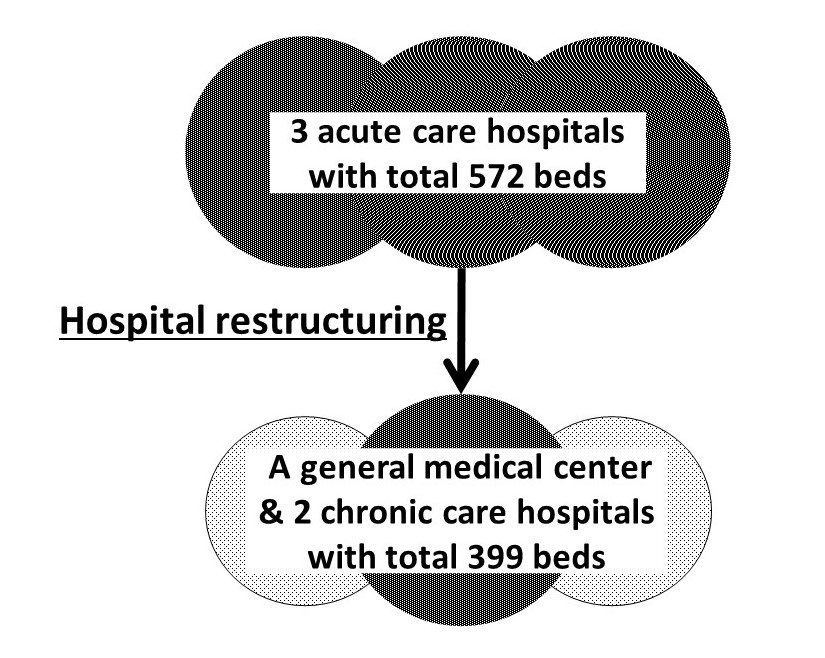

Supplement: Supplementary file 1 — Supplementary Material 1: Supplementary Fig. 1. Hospital restructuring in the secondary medical service area. [file 12913_2026_14388_MOESM1_ESM.jpg]
