# Supplementary material for: Impact of public hospital restructuring on the admission of elderly residents in Japan: a regional population-based study
Source: BMC Health Serv Res. 2026 Apr 2;26:686. doi: 10.1186/s12913-026-14388-3 (PMC13169532; doi:10.1186/s12913-026-14388-3)
Supplement: Supplementary file 2 — Supplementary Material 2 [file 12913_2026_14388_MOESM2_ESM.docx]

Supplemental Table 1. Background information on the SMSA and the prefecture.

|  | SMSA | Prefecture |
| --- | --- | --- |
| Population (%) |  |  |
| Overall | 81,041 (100) | 1,405,453 (100) |
| 15 – 64 years | 46,480 (57.4) | 865,578 (61.6) |
| ≥65 years | 26,614 (32.8) | 359,044 (25.5) |
| Number of hospitals | 4 | 75 |
| Number of hospital beds | 710 | 16,468 |

Abbreviations: SMSA, secondary medical service area.

Supplemental Table 2. Composition of general population aged ≥65 years in the SMSA during the study period.

|  | Pre-restructuring period (3 years) | | Post-restructuring period (4 years) | |  |
| --- | --- | --- | --- | --- | --- |
|  | First year | Last year | First year | Last year | |
| Overall population aged ≥65 years | 26,614 | 27,199 | 27,479 | 27,790 | |
| Age group, n (%) |  |  |  |  | |
| 65–69 years | 5,608 (21.1) | 6,323 (23.2) | 6,998 (25.5) | 6,926 (24.9) | |
| 70–74 years | 5,578 (21.0) | 5,659 (20.8) | 5,249 (19.1) | 5,498 (19.8) | |
| 75–79 years | 5,628 (21.1) | 5,249 (19.3) | 5,158 (18.8) | 5,101 (18.4) | |
| ≥80 years | 9,802 (36.8) | 9,968 (36.6) | 10,074 (36.7) | 10,265 (36.9) | |
| Female, n (%) | 15,538 (58.4) | 15,771 (58.0) | 15,888 (57.8) | 15,981 (57.5) | |

Abbreviations: SMSA, secondary medical service area.
